# Socioecological influences on concussion reporting by NCAA Division 1 athletes in high-risk sports

**DOI:** 10.1371/journal.pone.0215424

**Published:** 2019-05-08

**Authors:** Steven R. Corman, Bradley J. Adame, Jiun-Yi Tsai, Scott W. Ruston, Joshua S. Beaumont, Jessica K. Kamrath, Yanqin Liu, Karlee A. Posteher, Rikki Tremblay, Lisa J. van Raalte

**Affiliations:** 1 Center for Strategic Communication, Hugh Downs School of Human Communication, Arizona State University, Tempe, Arizona, United States of America; 2 School of Communication, Northern Arizona University, Flagstaff, Arizona, United States of America; 3 Global Security Initiative, Arizona State University, Tempe, Arizona, United States of America; 4 College of Health Solutions, Arizona State University, Tempe, Arizona, United States of America; 5 Sun Devil Athletics, Arizona State University, Tempe, Arizona, United States of America; 6 Department of Human Communication Studies, California State University at Fullerton, Fullerton, California, United States of America; 7 College of Business, California State University Monterey Bay, Seaside, California, United States of America; 8 Department of Communication Studies, Sam Houston State University, Huntsville, Texas, United States of America; Public Library of Science, UNITED KINGDOM

## Abstract

Concussion among athletes is an issue of growing concern, with efforts underway to improve detection, diagnosis, and treatment. Success depends on communication by athletes, as brain-related symptoms are often not outwardly visible. Education programs to increase reporting behavior have not been successful to date. In accordance with the socioecological approach to health, we argue that multiple levels of influence on student athletes must be addressed, and report a multi-dimensional, mixed-methods research project conducted to identify possible points of intervention into changing the culture of concussion-injury reporting among collegiate athletes. Using quantitative, qualitative and interpretive methods, we examine the individual-level *vested interests* athletes have in reporting or not reporting concussion symptoms, and how these interests interact with community-level *team culture* and *interpersonal relationships*, and social-level *cultural narratives* to influence concussion-reporting behavior. Our findings confirm the viability of this approach, identifying immediacy, separation of responsibility and pain-enduring story systems as particularly salient elements. We conclude that competing performance versus safety value structures, reflected in cultural narratives and team culture, create mixed-messages for athletes, which are resolved in favor of performance because athletes perceive concussion injuries to be of low immediacy.

## Introduction

Awareness of concussions as a problem in college sports has been on the rise in recent years. Though playing sports—particularly those involving intentional contact—has always carried a risk of concussion, a growing list of NFL players diagnosed with chronic traumatic encephalopathy (CTE; including sufferers who have committed suicide [1,2]) has dramatically raised concern about the issue. As public consciousness has grown, medical research into the effects of concussion has increased [3]. This has caused growing worry by parents [4,5], a corresponding push by schools, athletic associations, and city/state governments to establish safety and treatment protocols, and alarm in some quarters that the long-term future of youth contact sports is at risk [6]. For example, recent reports show that participation in high school football is down in 40 U.S. states [7].

Concussions present a unique challenge as an athletic injury. In some cases, there are visible outwards signs such as a blank stare, a balance disturbance, or a loss of consciousness. However, in most cases symptoms are internal, such as difficulty thinking clearly, blurry vision, sensitivity to light and/or noise, and fatigue. Because these are solely perceived by the athlete, communication becomes a fundamental issue: Athletes must recognize and report symptoms for diagnosis and treatment to be effective. Embedded within athletes’ reporting decisions are elements of concussion knowledge, attitude and self-identity, attitudes of peers and coaches, and the messages communicated by the broader culture.

To date, intervention efforts to promote reporting have focused on educating athletes about signs of brain injury and associated risks, and the importance of reporting symptoms [8,9]. This approach assumes that if athletes have adequate knowledge and tools, their health interests will manifest themselves in protective behavior. One study found that increased student athlete knowledge about concussion was indeed positively associated with concussion reporting, and this may have resulted in more recognition of concussion signs and symptoms among student athletes [10]. But another showed that, although athletes knew that playing with concussive symptoms is dangerous, they did not want to stop playing and report symptoms because they might be removed from play [11]. On balance, research indicates that simple concussion education is not effective [12]. Meanwhile, potential concussions continue to go unreported [13]. We argue that current education programs aimed at improving knowledge and encouraging self-reporting fail because they overlook differences between athletes/sports, competing interests athletes have, and effects of the organizational/cultural context in which they make health decisions.

Public health research has recognized the importance of factors beyond personal attitudes and beliefs for years[14]. The *socioecological framework* emphasizes that public health challenges involve a complex interplay between factors at multiple levels. The hierarchies of influence have been explained in various ways [15–17], but there is general consensus that individual, interpersonal, group/community, and social/cultural levels all have important effects on motivating health behaviors. The factors at these levels interact with and affect each other, and have been successfully applied to inform multi-level interventions for behavior change [17].

Kerr and colleagues [18] argue that concussion reporting research is inappropriately biased because it focuses exclusively "intra-personal and inter-personal level factors" (p.1020). Register-Mihalik and colleagues [16] provide an overview of the potential for improving concussion prevention and education strategies through the socioecological framework. To realize this potential, additional research is needed to explore the full range of the socially embedded levels in the contexts of head safety and concussion reporting.

As part of a 3-year study sponsored by the NCAA, we address the research gap through a socio-ecological approach considering individual-level *vested interests*, community-level *interpersonal relationships* and *organizational culture*, and social-level *cultural narratives*, and explore how these dimensions interact to influence reporting decisions by NCAA Division 1 athletes in high-risk sports. This paper lays out the theoretical and methodological foundation of this project, and provides empirical results of its first phase, comprised of studies of the individual/community aspects of concussion reporting, with integration of the findings in the socioecological framework at the end. Through the rest of the paper, we refer to severe head impacts (SHIs) rather than concussions to account for possible injurious blows to the head that occur but are not reported and/or diagnosed as concussions. In the next three sections, we review the proposed factors and their potential influence on athletes’ decisions about whether to report concussion symptoms.

### Individual-level: Vested interests

Attitudes and psychological states exert influence on communicative decisions [19]. As with other behavioral and communicative decisions, the choice to report concussive symptoms is influenced by a constellation of competing individual-level attitudes and cognitions. To measure these competing attitudes, beliefs, and cognitions relevant to brain health and SHI-based risk perception, this research uses Vested Interest theory (VI). It examines multiple factors that mediate the attitude-behavior relationship, and has demonstrated efficacy in risk perception and message-based interventions designed to enhance risk perception [20]. Understanding the vestedness of SHI-based risk perceptions and their influences should illuminate the intention formation and decision-making behaviors associated with brain health.

Research in other risk contexts supports this observation. Meta-analytic research has demonstrated that perceived risk is positively correlated with protective behavior and that increasing risk appraisal has a positive influence on both intention and behavior [21]. Sjoberg argues risk perception is a crucial component of successful campaigns; following this logic, we argue that educational programs meant to inspire self-protective action must also account for perceived risk. Perceived risk strongly influences adoption of self-protective behaviors and is crucial to understanding decisions to adopt health behaviors [22].

Behavioral intention, as an outcome, is potentially influenced by several precursors including perceived risk, as well as behavior-relevant attitudes, beliefs, and perceived social pressure [23,24]. We argue that VI’s attention to the associations between attitudes, beliefs, and behavioral decision-making offers a valuable method for examining individual level factors and their interrelationship with organizational and cultural factors. While VI focuses on individual-level attitudes and cognitions, it importantly makes no assumptions of rational decision making, and allows for the influence of organizational and cultural effects on the attitude-behavior relationship [25,26]. Measuring behavioral intentions without first understanding and accommodating perceived risk, is of limited value [27–29].

To predict perceived risk, we apply vested interest theory’s six distinct belief variables: *stake*, *salience*, *certainty*, *immediacy*, *self-efficacy* and *response-efficacy*. Together they mediate the relationship between attitudes and associated behaviors. Each element measures a specific evaluative dimension of attitudes, objects, and consequences; each aspect is theorized to exist in all individuals [30]. The variables, together, mediate the relationship between attitudes and complementary behaviors. When an individual evaluates each dimension at a high level, they are argued to be highly vested; alternatively, if one or more factor is judged at a low level, vestedness is reduced and the predictive value of attitudes is attenuated [25,26].

*Stake* is a global variable that characterizes an individual’s perceived involvement in an issue. Past research has characterized stake as a demographic variable, where individuals as a function of their decisions or present context, have an interest in the gain/loss consequences associated with that situation; moreover, one can have a stake in a given context without necessarily being aware [31]. In the present research, athletes in our sample who participate in contact and collision sports have a stake in SHI-related issues, by definition, because participation carries an elevated risk of suffering a SHI.

*Salience* describes the cognitive accessibility of related thoughts, attitudes, and behaviors. When an issue is salient, individuals can easily recall information, generate cognitions, and report attitudes that connect with the issue in question. Sivacek and Crano [32] argue that salience is a function of personal experience in a given environment, in this case being a member of a team and university. Athletes for whom SHIs are highly salient are likely ones who have been exposed to information, stories, and experiences that highlight the general subject.

*Certainty* describes the perceived probability of action and consequences related to the attitude-object. When the consequences associated with performing (or not performing) an attitude-relevant behavior are certain, the probability that individual will engage in that behavior is enhanced; consequences judged to be uncertain likely diminish that probability [20,25]. Regarding SHIs, athletes are likely to make judgements about the relative certainty that SHI-related consequences will occur. This level of perceived probability then mediates their reporting decisions and self-protective behaviors.

*Immediacy* is a temporal evaluation, describing how perceptually far in the future people believe actions may be warranted or consequences may be suffered. Environments where consequences of action are perceived to be near in time will tend facilitate attitudinally consistent behavior. Likewise, consequences perceived to be temporally far in the future reduce perceptions of vestedness, therefore attenuating the attitude-behavior link [20,25].

*Self-efficacy* is the athlete’s belief in her/his ability to act in a meaningful way or affect change. Within VI, self-efficacy follows Bandura’s original conception [33–35], occurring as both a trait and a state variable where individuals typically have a trait-based criterion level of self-efficacy that influences their interactions on a global level, and state-based criterion levels of self-efficacy that co-vary with context [36]. In the present research, we characterize self-efficacy as the athletes’ perceptions of their personal ability to manage their SHI-related risk.

Finally, *response-efficacy* is the belief that a personal response choice will be effective. Response-efficacy is adapted from fear appeal research that has shown the concept to useful in explaining behavioral decisions [31,37]. Recent VI-based research has recognized that when individuals are presented with a given set of response options, they are judged, in part, on their perceived effectiveness [31]. Athletes, because their athletic participation is influenced by multiple levels of organizational oversight, are presented with several response options if they suffer a SHI.

When each of the above vestedness factors is perceived to be high, pro-reporting attitudes should reliably predict behaviors; however, when one or more factors is low, the predictive strength is reduced [20,25,32]. In risk contexts, the VI model predicts attitudes connected to perceived risk and susceptibility to negative outcomes [20,38]. Understanding how vestedness frames risk decisions can therefore facilitate cultural change by enhancing risk perception and individual protective behaviors [20].

RQ1: What are athletes’ vested interests with respect to reporting and not reporting SHIs, and how do these differ by sport and sex?

### Interpersonal and community-level: Organizational culture

Recent studies have begun to address the prominent influence of supra-individual considerations on athlete reporting behavior [16]. Interpersonal relationships in the team setting with coaches [39], Athletic Trainers (ATs) [40], and teammates [41] can affect an athlete’s willingness to report head injuries and play when injured. Community-level (team) factors encompass organizational structure, medical staffing, the physical playing environment of a team, and formal concussion management policies and procedures. For example, most recent evidence has shown that organizational structures at schools where athletic departments supervise sports medicine departments might lead ATs and team physicians to report greater pressure from coaches to prematurely return athletes to play after a concussion [42].

At the interpersonal level, we argue that internal communication between key stakeholders—coaches, team physicians, ATs, and teammates—forms the basis of perceived norms regarding SHI reporting, which in turn can amplify or reduce the influence of VI variables on an athlete’s health decision making. As noted in the organizational sensemaking literature,

[c]ommunication is an ongoing process of making sense of the circumstances in which people collectively find themselves and of the events that affect them. This sensemaking, takes place in interactive talk and draws on the resources of language in order to formulate and exchange through talk symbolically encoded representations of these circumstances. [43] (p. 143)

According to Schein, organizational culture is the pattern of shared beliefs that guide group members’ perceptions, feelings, and actions, and is reflected at three levels: artifacts, espoused values, and basic assumptions [44]. *Artifacts* include visible products, such as language that while easy to observe may often be ambiguous and difficult to decipher [44]. Artifacts may be tangible, as in the case of posters and murals in team spaces exhorting team values, celebrating high profile alumni, or listing the symptoms of a concussion. Artifacts can also take the form of team slogans, theme songs, and rituals. *Espoused values* are evaluative standards created through a process of group learning that reflect shared values; over time these can become shared assumptions that are basic to the group culture [44]. Espoused values and norms are less visible than artifacts and are the most powerful influences on desired behaviors, such as organizational structures and internal communication guiding concussion prevention and management. *Basic assumptions* evolve as a solution to a problem and are repeated over and over again, and are constructed as a social reality [44].

Norms and basic assumptions of the organization can have a powerful influence on individual members (athletes, ATs, medical professionals, and coaching staff). Pressure to conform can alter attitude/behavior expressions an individual would otherwise display [45–47]. Recent studies have established the importance of coaches’ or ATs’ roles in concussion reporting [40,48]. Despite this, little is known about how athletes’ attitudes and norms for reporting SHIs are shaped by interpersonal-level and community-level factors.

RQ2: How do community-level factors and interpersonal communication dynamics shaped by organizational (team) culture influence athletes’ SHI reporting behavior?

### Social-level: Cultural narrative

At the social level of influences on SHI reporting are *cultural narratives*. Narrative is both simultaneously a cognitive process of making sense of the world around us [49], and a socio-cultural phenomenon identified as a system of stories sharing a common structure of desire/conflict, sequential event trajectory, and resolution. These aspects of narrative shape identity and behavior [50] (p. 107). Since cultural narratives both embody specific values and provide cognitive templates for comprehending contemporary events and situations [51], understanding the content, structure and components of the narratives of sports culture illuminates societal levels of influence.

For example, sports media have long celebrated stories of toughness, and promoted a culture of risk [52]. Recently, the tragic story of Junior Seau and similar stories have introduced a narrative of caution and consequence into the cultural conversation around head injuries and concussions. Narratives can highlight risk or elide risk and consequence in their celebration of toughness or other ideals, influencing perceived risk and shared basic assumptions among athlete communities.

*Narrative logic* is an important factor in understanding the influence of narrative on how individuals make sense of cause and effect, conceive consequences, and make decisions. Fisher regards humans as story-telling animals (*homo narrans*) who are heavily influenced by a *narrative* logic predicated on *coherence* (internally consistent story logic) and *fidelity* (congruence with stories already believed to be true by the individual) [53]. Thus, athletes may not accept a story told about the near-term consequences of concussion injury because the stories they already believe to be true place its debilitating effects in old age, allowing them to make narrative-rational decisions to favor performance over safety. Cultural narratives also execute ideological functions, including naturalizing of constructed behavior and universalizing of interests [54,55].

In another study [56] conducted under the same funded project supporting the study reported here, we described the cultural narrative system of high-risk college sports. We identified eleven relevant narratives, and singled-out five that likely have significant effects on concussion reporting decisions. Four of these were grouped into a *performance narrative* class. These included (a) play through pain, which suggests that athletes endure pain as heroic protagonists sacrificing their health for the betterment of the team, (b) making the big leagues, advancing the notion that hard work and perseverance will ultimately result in a professional athletic career, (c) commodification, which highlights how the professional sports industry treats players as commodities, and (d) the masculine warrior, in which the athlete protagonist embodies traits of masculinity and defeats an opponent through masculine-warrior behaviors. These four narratives are old and deeply institutionalized, and have short time horizons for resolution, often within a game or season.

In contrast, the need for safety narrative is new and not deeply institutionalized. It supports concussion reporting behavior and focuses on the long-term health of the athlete and his or her ability to continue playing. This narrative also has a longer time horizon, as debilitating health effects of concussion may not manifest themselves in the context of a game or season, but later in life.

Next, we describe quantitative and qualitative research that explains how individual and interpersonal/community factors influence concussion reporting, to round-out the socio-ecological model. In the discussion we combine this with the findings from the existing cultural narrative study to propose a model of how all three levels influence concussion reporting.

## Methods

### Common research setting and participants

This research was approved by the Arizona State University Institutional Review Board, STUDY00004049. Written informed consent was obtained for surveys, and oral informed consent was obtained for interviews. We addressed the research questions above using quantitative and qualitative methods examining two levels of socio-ecological factors. Individual-level factors were tested using quantitative surveys, and interpersonal/community-level factors were examined through qualitative interviews. However, they both share common features described here. Data were collected from Division 1 collegiate athletes participating in six NCAA-sanctioned sports that pose a high concussion risk, as identified by the NCAA Injury Surveillance Program [57]. Participants were solicited from eleven universities governed by a major athletic conference. Liaisons in athletic programs from each participating school assisted in recruiting participants. Random sampling was not practical because of time conflicts with play/practice schedules, so participants were recruited on an availability basis. Athletes were not prohibited from participating in both the survey and the interview; the survey was only open to athletes.

For the survey, the liaisons recruited male and female athletes in six sports: football, soccer, basketball, wrestling, field hockey, and lacrosse. While concussion incidence in basketball is not as high as some other sports, it was chosen because it is the only collision/contact sport with male and female teams at all the participating schools, facilitating sex-based comparisons. Recruiters had a goal of *n* = 50 athletes per school, distributed among the target sports. For organizational culture interviews, we focused on basketball and women’s soccer for this phase of data collection (Phase 2 will conduct interviews in the remaining sports). We aimed to recruit three student athletes, one coaching staff member, and one AT staff member per school per team. All participants were incentivized with gift cards for participation, with $20 for a completed survey, and $30 for a completed interview. Surveys and interview protocols were developed within standard human subjects guidelines and were approved by Arizona State University’s Institutional Review Board.

### Quantitative survey: Vested interests

#### Participants and procedure

Participants were solicited via the procedure described above and were directed to a web-based survey where they acknowledged informed consent and completed their participation. In total, 590 athletes clicked-through to the online survey. We excluded 189 responses because of incomplete surveys, duplicates, or evidence of response-sets. Athletes were asked to report their sex and university, the sport in which they participate, and the number of years they have participated. The athletes then responded to measures described below and were asked to report the number of diagnosed concussions and SHIs that were not diagnosed as concussions. The survey ended by asking athletes to report demographic information. Upon completion, athletes were compensated and thanked for their participation.

#### Measures

Athletes were asked to respond to scales measuring vestedness in attitudes related to their risk perception and decision-making related to suffering SHIs (see S3 Table). Vested Interest Scales were modified from natural hazard research to fit the present context; construction and validation of these scales, as well as the risk perception scale, is reported in existing publications [20,31,58]. We modified the Vested Interest Scales for the SHI context by substituting appropriate nouns and phrases into the items. Questions were targeted toward SHIs because a concussion is a formal diagnosis that requires communicative participation and self-reports of symptoms [59].

Consistent with their use in other research, each of the VI sub-scales and the risk perception scale consists of five items, with each group of five targeted toward one of VI’s specific sub component. Each VI sub-scale, as well as the perceived risk instrument consisted of five items, with a seven-point response set anchored by *strongly disagree–strongly* agree; higher values correspond to increased perceptions of vestedness or agreement, respectively. The scales were then pilot tested on a sample of Division 1 and club-level athletes participating in activities with varying levels of concussion risk at a Power-5 conference university. Pilot testing on a sample of n = 30 NCAA and club athletes at Arizona State University showed that the modified scales were reliable in the range 0.71 ≤ α ≤ 0.92, indicating that they were robust to modification to the SHI context. In the main study, the composite scales demonstrated acceptable reliability: α_salience_ = 0.77, α_certainty_ = 0.85, α_immediacy_ = 0.85, α_self-efficacy_ = 0.80, and α_response-efficacy_ = 0.92. Athletes were also asked to report their perceived risk of suffering consequences related to SHIs (α = 0.87).

#### Analysis

Our analytic approach had two goals to explore overall levels of athlete’s perceived vestedness and how these might vary by sport, and to understand the viability of the VI model for explaining and predicting variance in perceived risks associated with SHIs. Our analysis used analysis of variance and regression approaches. We first performed a one-way ANOVA to test for significant mean differences between the VI subscale data. Next, because our data is structured with pre-existing groups (sport and sex), and because each sport varies in its objective SHI risk, we performed MANOVA analyses, using these groups as predictor variables. We then performed a regression analyses to verify the predictive capacity of the VI variables. Finally, to examine potential sex differences between athletes in the same sport (basketball), we performed independent samples *t-*tests.

### Qualitative interviews: Organizational culture

#### Participants

We conducted the interviews with athletes, ATs, and coaches in English using a standardized protocol during June 2016 to January 2017. Semi-structured interviews were conducted face-to-face, by telephone, or via Skype, and were audio-recorded with participants’ oral informed consent. Participants were from men’s and women’s basketball teams, and women’s soccer teams. In total, 90 interviews (11 coaching staff interviews, 23 AT interviews, and 56 athlete interviews) were completed averaging 29.2 minutes in length. All verbatim transcripts were obtained through a professional transcription vendor and entered into *NVivo 11* to assist in coding and analysis.

Based on formal interview guides, athletes were asked to tell a story about a time they experienced a severe head impact or witnessed a teammate who experienced a severe head impact with follow-up questions about the incident, such as “What were you thinking and feeling at the time of the incident” or “Did you (or your teammate) feel pressure to stay in the game or practice?” Athletes were also asked to discuss team slogans to better understand organizational values and norms beyond narratives of SHI experiences. ATs were asked to describe the organizational structure, concussion education, and processes involved when a player suffers a SHI. We also wanted to understand any barriers or challenges within formal protocols and processes through questions such as “What barriers do you face when diagnosing an athlete with a concussion” and “Can you tell me about some of the challenges you face when removing an athlete from participation.” Lastly, interviews with coaching staff provide insights into organizational culture, team identity and coaching philosophies in connection with concussion policies and education. For example, we asked coaches to “describe the identity of their team” and “what characteristics do you value in players” in addition to asking coaches about concussion education, processes, and protocols and their role in each.

#### Analysis

We utilized Schein’s organizational culture framework as a lens to guide our analysis and serve as sensitizing concepts that framed our data [60,61]. We used the constant comparative method [62] in our primary cycle coding to identify elements of Schein’s framework in eleven initial codes (see S1 Table and S2 Table). A code can be expressed in a single word, a phrase, a sentence, or a paragraph; team members assigned codes in multiple categories to text blocks of any size. Schein’s framework, previous literature on concussion management, and preliminary interview results provided us with a provisional coding structure [63] that guided several rounds of iterative analyses to build consensus and refine definitions. To validate codebook development, all team members annotated the same five randomly selected transcripts using the initial framework. Deliberating the first round of analysis lead us to believe that majority of codes derived from Schein’s framework were valid and applicable in this study context as there is no prior research examining organization culture and concussion reporting. A second round of open coding with new 30 interview transcripts enabled further development of inductive codes essential in determining SHI reporting behavior. The iterative refinement of codes was traced weekly and continued until code definitions began to stabilize with no new changes. The iterative steps taken to validate codes allowed us to examine the multifaceted influences of organizational and interpersonal factors in concussion management. More important, it generated requisite variety, an important criteria to ensure theoretical rigor and data richness in qualitative research [64,65]. Next we conducted a thematic analysis for identifying key themes and emergent patterns [63,66] within each of the existing codes. We identified themes that referenced communication processes within the organizational culture in the context of congruence/incongruence between athlete and staff interviews. Following analysis of 90 interviews covering a diverse range of interviewees’ demographics and injury context, we deemed that data saturation was achieved because themes were adequately exemplified in the data and robust enough to gain a comprehensive understanding of the research question [67]. Additionally, we constantly checked with an experienced AT team member to justify that key themes were robust to offer nuanced understanding of reporting behavior.

## Results

### Individual-level: Vested interests

Athletes included in the survey (*N* = 401) were 51.6% female, with an average age of 20.14 (*SD* = 1.60), and an average of 2.39 (*SD* = 1.29) years of collegiate education. Most participants reported their ethnicity as White (60.5%), with 18.0% reporting Black/African American, 10.0% reporting Hispanic/Latino/a, 3.0% reporting Asian, 2.0% reporting Native Hawaiian/Pacific Islander, 0.8% American Indian/Alaskan Native, and 5.8% reporting Other. Male and female soccer players comprised the largest overall group of participants (*n* = 139), followed by male and female basketball players (*n* = 89), male football players (*n* = 86), male wrestlers (*n* = 48), female field hockey players (*n* = 2), and female lacrosse athletes (*n* = 37). For the purposes of these analyses, field hockey and lacrosse athletes were combined into a single group due to the similarity of the sports. Information on scholarship status, participation, and head trauma are shown in Table 1.

**Table 1:** Scholarship, participation, and head trauma by sport.

Variable	All	Football	Wrestling	Soccer	Basketball	FH & Lacrosse
Scholarship Status						
Full ride	47.6%	65.5%	8.0%	32.6%	93.4%	2.6%
Partial	36.7%	1.1%	60.0%	58.9%	0.0%	94.9%
None	15.7%	33.3%	32.0%	8.5%	6.6%	2.6%
Participation^a^						
Sport	12.37(3.77)	9.7(3.24)	11.31(3.85)	14.79(2.84)	12.24(3.77)	11.10(2.45)
University	2.12(1.14)	2.18(1.24)	2.75(1.85)	2.14(1.45)	1.77(1.28)	2.08(1.20)
Head trauma ^b^						
Concussion, diagnosed	35.7% 0.61(1.03)	43.7% 0.78(1.28)	35.4% 0.58(1.00)	39.7% 0.71(1.06)	29.7% 0.38(0.70)	20.5% 0.33(0.81)
Possible concussion, undiagnosed	35.9% 0.87(1.70)	46.0% 1.38(2.57)	47.9% 1.16(1.64)	37.6% 0.88(1.49)	23.1% 0.38(0.95)	25.6% 0.36(0.66)

^a^Entries are mean(std. deviation).

^b^Entries are the percentage of participants reporting such trauma, and the mean and std. deviation of the number reported incidents for those participants.

**Fig 1:**
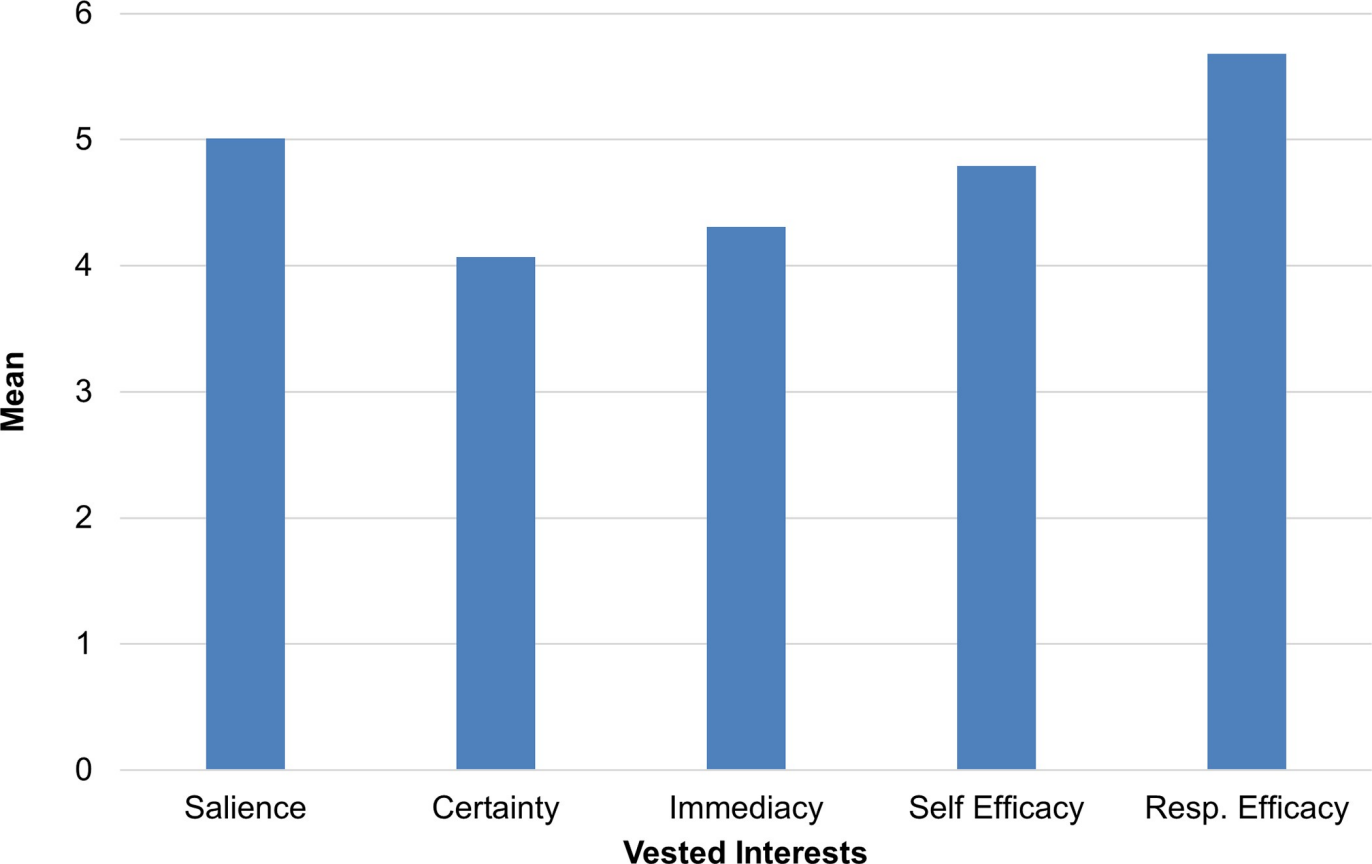
Means of vested interests variables for all participants.

Table 2 shows the means and standard deviations for all athletes in each of the sports included in this study; Table 3 shows the sample moments and bivariate correlations for the VI variables and perceived risk. Means for the VI variables are significantly different across all participants and sports, *F*(4, 2000) = 135.69, p < .001, and are shown graphically in Fig 1. Certainty has the lowest mean while the second lowest value is for immediacy.

**Table 2:** Means and standard deviations for vested interests.

Sport	n	Salience	Certainty	Immedi-acy	Self-Efficacy	Response-Efficacy	Perceived Risk
All	401	5.01(1.00)	4.07(1.22)	4.31(1.23)	4.79(0.97)	5.67(0.97)	4.92(1.18)
Football	86	5.25(1.08)	4.30(1.33)	4.30(1.09)	4.63(1.10)	5.37(1.20)	5.01(1.16)
Wrestling	48	4.96(0.90)	3.82(1.09)	3.98(1.14)	5.02(0.83)	5.86(0.74)	4.86(1.17)
Soccer	139	5.11(0.95)	4.14(1.18)	4.44(1.15)	4.91(0.93)	5.85(0.85)	5.08(1.10)
Basketball	89	4.70(1.01)	3.86(1.31)	4.33(1.23)	4.67(0.93)	5.61(0.97)	4.55(1.23)
FH/Lacrosse	39	4.94(0.96)	4.12(0.99)	4.31(1.33)	4.74(0.95)	5.61(0.88)	5.08(1.20)

Entries are mean(std. deviation)

**Table 3:** Bivariate Correlation Analysis.

Variables		*M(SD)*	1.	2.	3.	4.	5.
**1.**	**Salience**	5.01(1.00)	–				
**2.**	**Certainty**	4.07(1.22)	.35*	–			
**3.**	**Immediacy**	4.31(1.23)	.35*	.47*	–		
**4.**	**Self-Efficacy**	4.79(0.97)	.11**	-.02	.00	–	
**5.**	**Response-Efficacy**	5.67(0.97)	.20*	.01	.06	.36*	–
**6.**	**Perceived Risk**	4.92(1.18)	.47*	.46*	.45*	-.09	.02

N = 401

*p < .001

**p < .05

To assess differences between categorical variables, we performed a MANOVA with sport, sex, and scholarship status as the independent variables and the VI factors as dependent variables. Scholarship status was not a significant predictor (Wilks’ λ = .982, *p* = .723) and was removed from the model. The revised omnibus test was significant for both sport: Wilks’ λ = .894, *F*(5, 20) = 2.23, *p* = .001, η^2^ = .03; and sex: Wilks’ λ = .956, *F*(5, 20) = 3.61, *p* = .003, η^2^ = .04; the interaction between sport and sex was not statistically significant (*p* = .438). Examination of the univariate results for sport show the significance of the overall model is being driven by perceptions in salience, *F*(4, 400) = 3.66, *p* = .006, η^2^ = .04, certainty, *F*(4, 400) = 2.67, *p* = .032, η^2^ = .03, and response-efficacy, *F*(4, 400) = 4.16, *p* = .003, η^2^ = .04. Self-efficacy, *F*(4, 400) = 2.02, *p* = .092, η^2^ = .02, approaches significance and accounts for some of the variance in the model. For sex, the univariate results show certainty, *F*(1, 400) = 4.63, *p* = .032, η^2^ = .01, and immediacy, *F*(1, 400) = 16.53, *p* < .001, η^2^ = .04. The results for immediacy are interesting; examination of the means shows that women perceive higher levels of short-term threats from concussions than men, but these perceptions are independent of their sport.

We also measured perceived risk of consequences from head impacts, and as a test of the VI model’s risk prediction capability, regressed each of the elements of vestedness on perceived risk, controlling for the influences of sport sex, and scholarship status. Again, scholarship status was not a significant factor (*p* = .61) and was removed from the model. Together, sport and sex account for 3% (*R*^*2*^_*adj*_) of the variance in perceived risk, *F*(2, 397) = 6.60, *p* = .002. The VI variables model, *F*(5, 392) = 45.77, *p* < .001, predicts 38% (*R*^*2*^_*adj*_) of the variance in perceived risk. See Table 4 for regression coefficients.

**Table 4:** Regression analysis for perceptions of risk.

Variable	B	SE	r	t-value
Model 1				
Constant	5.01*	0.20	–	
Sport	0.01**	0.03	0.15	2.95
Sex	-.317**	0.12	-0.14	-2.67
Model 2				
Constant	2.14*	0.41	–	–
Salience	0.38*	0.05	0.33	7.27
Certainty	0.22*	0.04	0.22	4.86
Immediacy	0.22*	0.05	0.23	4.82
Self-Efficacy	-0.14**	0.05	-0.12	-2.78
Response-Efficacy	-0.04	0.05	-0.03	-0.73

N = 401, R^2^ = .62, adjusted R^2^ = .38, *F*(5, 399) = 35.62, p < .001

*p < .001

**p < .01

Finally, to gain a more nuanced understanding of the role of sex, we separately analyzed the data for differences in vestedness based on sex. This analysis focused on basketball players because that sport has men’s and women’s teams at all participating universities, and both sports provide opportunities for post-collegiate/professional play. To discover potential within-sport, cross-sex differences we compared male (*n* = 34) and female (*n* = 55) basketball players with each of the elements of vestedness and perceived risk. Significant differences emerged for immediacy *t*(87) = 2.67, *p* = .009, *d* = 0.56 (males: *M* = 3.91; *SD* = 1.40; females: *M* = 4.60; *SD* = 1.04), and perceived risk *t*(87) = 2.60, *p* = .011, *d* = 0.55 (males: *M* = *4*.*14*; *SD* = 1.40; females: *M* = 4.82; *SD* = 1.06). Examination of the means shows that males perceive significantly lower levels of immediacy than females, indicating that male basketball players perceive the consequences of SHIs to be farther in the future than do their female counterparts. Means for perceived risk show a similar pattern in which male basketball players perceive significantly lower risk from SHI than do female basketball players.

### Interpersonal and community level: Organizational culture

We found athletes’ reporting decisions are shaped by interpersonal-level and community-level factors. In what follows, we report three emergent themes addressing a key role of organizational culture: perceived effectiveness of concussion education, separation of responsibility, and influence of internal communication dynamics on self-reporting.

#### Perceived effectiveness of concussion education

ATs typically assume responsibility for educational efforts. ATs furnish information to athletes and coaches regarding concussion in educational presentations and cover the most recent rules and regulations. As one student athlete recalled,

I mean also our trainer makes us aware of it. In the beginning of the season, he gives us as coaches just an educational reading that we read on concussions and all the stuff that’s going on. He’s the main source.

This example illustrates ATs’ initiating role in creating awareness among players; they also educate the coaching staff. One AT noted, “Not only do we have meetings with the student athletes, but we also have the individual meetings with the coaches where we go through our return to play protocol.”

The education delivery format includes in-person educational sessions, videos, and written documents (e.g. NCAA fact sheets and flyers). ATs provide student athletes general health information and concussion-specific knowledge, like signs and symptoms. Many times, concussion education occurs during sessions that are informational in nature and include an array of information beyond concussions, such as appropriate behavior in the training room, how and when to get treatment, or other general health information. In these informal sessions, many ATs emphasize building a rapport with players not only to encourage self-reporting but also to better detect symptoms when student athletes encounter SHIs. In rare cases, some ATs make further commitments and give team-based talks encouraging concussion reporting among teammates and creating a supportive team culture to highlight the mutual responsibility for taking care of each other. One female athlete elaborated on interaction with her AT:

She told us that if one our teammates was susceptible, if we thought that one of our teammates had a concussion, then as a teammate, we have the responsibility of checking up on them, and if they don’t wanna tell the trainer, then telling the trainer. Ultimately, we care about the health of our teammates.

Most coaching staff and ATs believe that their educational materials are informative; however, student athletes tend to say that educational materials (especially written documents) are not memorable. As one female athlete said, “I vaguely remember something being done about concussions, but only very briefly, during a compliance meeting.” Their recollections of concussion education fail to relay a depth of content-specific knowledge beyond remembering signing documents and knowing concussion is serious matter. One male player noted: “They gave us basic information, such as if you ever feel—if you ever get hit and you feel your equilibrium is off or something is seriously wrong as far as you can’t recollect different things, like it’s hurting you.” Another player reflected: “I don’t know much about concussions. I just know that they’re—they could be life threatening, and they are serious. I don’t know the proper care for them once if you do receive a concussion.” Moreover, education is not offered frequently. Many student athletes report that the concussion education is a one-time or once-a-year process. One athlete said, “people feel like it’s a one-time thing or something like that, but I don’t think it’s given to us enough on a regular basis.” The lack of repetition may impede student athletes’ understanding of significant risks and existing protocol of concussion management [68].

Additionally, most coaches maintain separation from the concussion education process and cede that responsibility to ATs. As one coaching staff member explains, “Our athletic trainer talks to the girls about it a lot. They have to do their ImPACT testing before we start. They know why they’re taking it so that if something was to happen and they do need to get evaluated that they understand what side of the scale they're on in terms of how severe the injury is. Honestly we don’t talk about it a whole lot in terms of verbal conversations except amongst us as a staff” (ImPACT is a protocol for detecting and managing concussions).

#### Separation of management responsibility

As explained above, ATs are responsible for administering concussion protocols, making diagnosis decisions, and actively building trusting relationships with coaches and athletes. While no coaches report intending to risk the long-term health of their athletes, they do not view concussion education and prevention as their primary responsibility. They receive information and updates from ATs, and sign acknowledgments of standard concussion protocol training. The separation of concussion management responsibilities reduces the opportunities for coaching staff to support educational efforts. Many ATs prefer that coaches’ involvement in internal communication processes be limited to listening and asking questions. Most ATs describe themselves as fortunate that coaches trust them to “do their job.” One AT pointed out, "I think I am fortunate where my coach is, I don’t wanna say hands-off, but usually if I say, 'hey this kid is out' he is like 'Okay.‴ One AT discusses the relationship with the coaching staff in saying,

The head coach lets me do my thing. Doesn’t meddle at all. In fact, doesn’t even wanna know the details. He wants to know enough that he can talk to the kid and talk to the parents, and talk to the media maybe, but other than that, everything is my decision, everything is what I say goes. Never been questioned on anything at all.

In these examples, we can see how the separation of responsibility is reinforced through the organizational structure and daily interaction between ATs and coaching staff, further shaping the most coaches’ hands-off approaches left to medical professionals.

These internal communication practices create a structural separation of responsibility, limiting coaches’ roles in promoting athletes’ SHI safety. This amounts to a firewall of sorts, where coaches focus on play and performance and ATs focus on SHI safety. A women’s soccer coach expressed “It’s all about the doctors and the trainer. I don't get involved in it. I think it's such a touchy subject.” This representative quotation illustrates coaches’ prioritization of team performance as their main job responsibility and their belief that remaining “hands-off” is in the best interests of athlete safety.

#### Influence of internal communication dynamics

Athletes engage in a cost-benefit analysis when deciding whether to report a SHI, such as the context of the injury (e.g. significance of the event; competition vs. practice; timing in the season), being the target of disappointment from coaches and teammates, potential attributions and blame from coaches and teammates (e.g. failing the team; being perceived as faking symptoms; mental weakness), and threats to their position and/or standing on the team. For example, a women’s soccer player said:

I was scared to tell the coaches because I didn’t know if they would believe me. With like a broken ankle, you can see it. With a concussion, it is based off your words. This is what happened. I was kinda nervous to tell them, for them to get mad at me, which was not smart.

Notably, interviews with both athletes and ATs suggest that effective diagnosis and treatment depends on self-reporting by athletes and relationship building. While ATs have diagnosis protocols, they are subject to error. Although ATs frequently witness impacts that require an athlete be removed from play or in some cases notice symptoms that lead to an athlete entering the concussion protocol, ATs must also rely on athlete’s trust and willingness to report collisions and symptoms in a timely manner. Trust between ATs and players is built on frequent everyday interaction, as described as “We honestly have more contact with them than the coaches do throughout the year just because the coaches can’t be at some weight-room workouts or they’re not in treatments and things like that.” ATs know that athletes may hide symptoms or avoid reporting, so they seek to build trusting relationships with their athletes. Establishing trust and a knowledge of a behavioral baseline facilitates ATs’ ability to detect when something is “not right.” One AT clearly illustrated this idea:

You know their mood when they get hurt. When I say, it’s easier to tell, cuz you know

they switch gears. It’s like, okay. I’m down. This hurts. Then you can also tell from the athlete, even though they’re done, okay, but they wanna play.

Through building a rapport with student-athletes, ATs can recognize symptoms even when an athlete does not report or tries to downplay the severity of symptoms. One AT, referencing having a good understanding of individual players needs and who they are, says,

I think that’s a really important aspect of what we do and why we work with the team, is because you get to know the personalities and get to know the kids on a personal level to where you know something is not right.

Another AT stresses this relationship in saying, “Part of that is you knowing the kid, and knowing what they look like, looking at their eyes…can you tell they’re not telling you truthfully?”

#### Basic assumptions

These findings indicate the presence of three relevant basic assumptions in the cultures of most of the teams studied. First, SHIs and concussions are the domain of ATs. These personnel conduct the education, spot and interact with athletes about questionable impacts, perform assessments, determine fitness to continue play, supervise treatment, and approve return to play. Conversely, most coaches are excluded from concussion prevention and management, including education, because their job is to manage the athletic performance of healthy players. A second basic assumption is that education consists of presenting players with standardized materials, usually once, at the beginning of the year, and documenting that they have received this training. It is not necessary to adapt these materials to different sports, or to assess the effectiveness of the educational interventions and make them more effective or memorable. Third, despite a general awareness of adverse impacts of SHIs, factors underlying one’s intention to report a SHI go well beyond the medical consequences upon which most concussion education is based.

## Discussion

Unlike other kinds of athletic injuries, concussions involve various symptoms, which may not be outwardly visible and might take time to manifest. Therefore, effective treatment relies on communication of symptoms by athletes to ATs. Above, we argued that the existing approach of educating athletes about concussion symptoms and risks is flawed because it relies on a one-size-fits-all approach and fails to consider factors other than individual attitudes. We proposed that vested interests, which mediate between attitudes and concordant behavior, are one potential influence on athletes’ SHI reporting behaviors. Drawing on a socioecological approach, we also noted that attitudes and vested interests are embedded in systems of organizational culture, which also play a role in athletes’ willingness to report severe head impacts (SHIs) and possible concussion symptoms. Both the individual and interpersonal/community levels are contextualized by the cultural narratives reported by Ruston, et al. [56]

To study these phenomena, we relied on an availability sample of athletes, ATs, and coaches. Typical of studies with similar recruitment strategies, there is possible self-selection bias for those who participated in the research, thereby leading to a variation of SHI experience with interviewers or with their teammates represented in our data. It is also possible that our liaisons biased recruiting toward athletes who would present a favorable impression of their teams. However, short of a mandate by the NCAA or other regulatory body for teams to participate in a random sample, we believe there is no feasible way to avoid availability samples, owing to the competing demands of athletes and teams to play, practice, and attend school.

Research question 1 asked: What are athletes’ vested interests with respect to reporting and not reporting SHIs, and how do these differ by sport and sex? Results indicated, first, that vested interests differ significantly by sport and sex, supporting our argument that educational efforts need to be tailored for different kinds of athletes. Certainty was the lowest of the VI elements, indicating that athletes are uncertain that they will suffer SHI consequences. Immediacy was the second lowest of the vested interests, indicating that athletes perceive negative consequences of severe head impacts as lying in the distant future. These results are concerning, because, as noted above, concussions also have negative health consequences that manifest in the short-term. Meanwhile, narratives of victory on the playing field, respect for toughness and sacrifice, and the redemptive value of athletic participation are all rendered in immediate terms and negative consequences are subordinated or elided. These more immediate concerns are likely to drive athlete reporting behavior. Results also showed that as athletes’ vested interests increase, their level of perceived risk increases, and with the regression model accounting 38% of the variance. This interpretation corresponds with other risk-based VI research [20,38], and suggests that increasing immediacy may increase protective behavior.

Research question 2 asked: How does organizational (team) culture influence athletes’ SHI reporting behavior? Our interviews revealed three important themes in organizational culture that bear on this question. First, all teams make standard efforts, led by ATs, to educate members about concussion symptoms, risks, and procedures. However, few coaches actively engage in the education process, education takes place once per year in most cases, and athletes do not find it memorable. Education to promote safety is neither frequent nor memorable, and is not emphasized by coaches, the formal team leaders. We see here a basic assumption that concussion education is treated as a formality rather than an ongoing communication effort to be reinforced, evaluated, and optimized to reshape a culture of encouraging early reporting and safety. Our findings do not suggest that coaches have no role in promoting head safety. For instance, many teach athletes techniques to avoid moves that would results in SHIs. But these are before-the-fact measures that are, again, focused on performance. We also note that there are some exceptions to the general findings above. For example, in one of the teams the coach took an active role in endorsing education. Another team produces its own training materials in video format. However, these were rare exceptions, and these basic assumptions stated above fairly characterize the teams we studied in general.

Second, most teams maintain a firewall separating coaches from decision-making about SHIs. While there is general agreement that coaches should not be involved such decision-making, an unintended consequence of their exclusion is their lack of involvement in reinforcing team norms and immediacy for reporting. It also establishes a formal structure in which coaches emphasize play and performance, while ATs emphasize safety, making athletes accountable to two different sets of conflicting values. Although we acknowledge that athletes’ health decisions should be left to medical professionals like team physicians and ATs, this separation lends itself to the unintended consequence of limiting coaches’ roles in endorsing concussion education, promoting a culture of safety, and having meaningful safety-related conversations with athletes.

Third, regarding internal communication dynamics, athletes calculate costs and benefits of SHI reporting by considering not only the health implications, but also the impact on their prospects as a member, impact on the team, and possible undesirable reactions from teammates and coaches. Perceived pressure to not report is internalized through social interactions within teammates and coaches (see also [48]). Athletes report being “scared” about how coaches will react and worry that others will not believe their reports. While it might be plausible that probing questions related to pressure to stay in the game or practice might lead participants to give desirable answers, 52% of athletes revealed that the common pressure came from themselves because of tough mentality, wanting to play to ensure team performance, and intense frustration being sidelined regardless of experiencing SHIs or witnessing teammates’ incidences. Factors contributing to athletes’ perceived self-pressure are consistent and independent of athletes’ diagnosed concussions or teammates’ SHIs. A previous conclusion suggests a basic assumption that engaging in concussion safety management and communication is the domain of ATs. Because of the reliance on self-report, ATs constantly acknowledge the importance of building a trusting relationship with athletes to optimize the potential of early identification when athletes do not want to seek medical care. Coaches are not to be involved in education, detection of symptoms, or decision-making, and instead are to focus solely on athletic performance Together, athletes’ diminishment of medical consequences is consistent with the lack of immediacy and the influence of institutionalized cultural narratives that valorize playing through pain and competing at any cost.

### Integration of socioecological levels

This study’s multi-dimensional approach illuminated relationships that would not have been evident in single method studies. A single study could have identified vested interests of athletes in SHI reporting, but would have lacked the context of the interpersonal, community, and cultural influences that frame their reporting decisions under low immediacy. Likewise, a study of organizational culture would have identified a firewall between coaches and ATs with respect to concussion reporting but could not have linked the divide to narrative systems with different levels of institutionalization.

**Fig 2:**
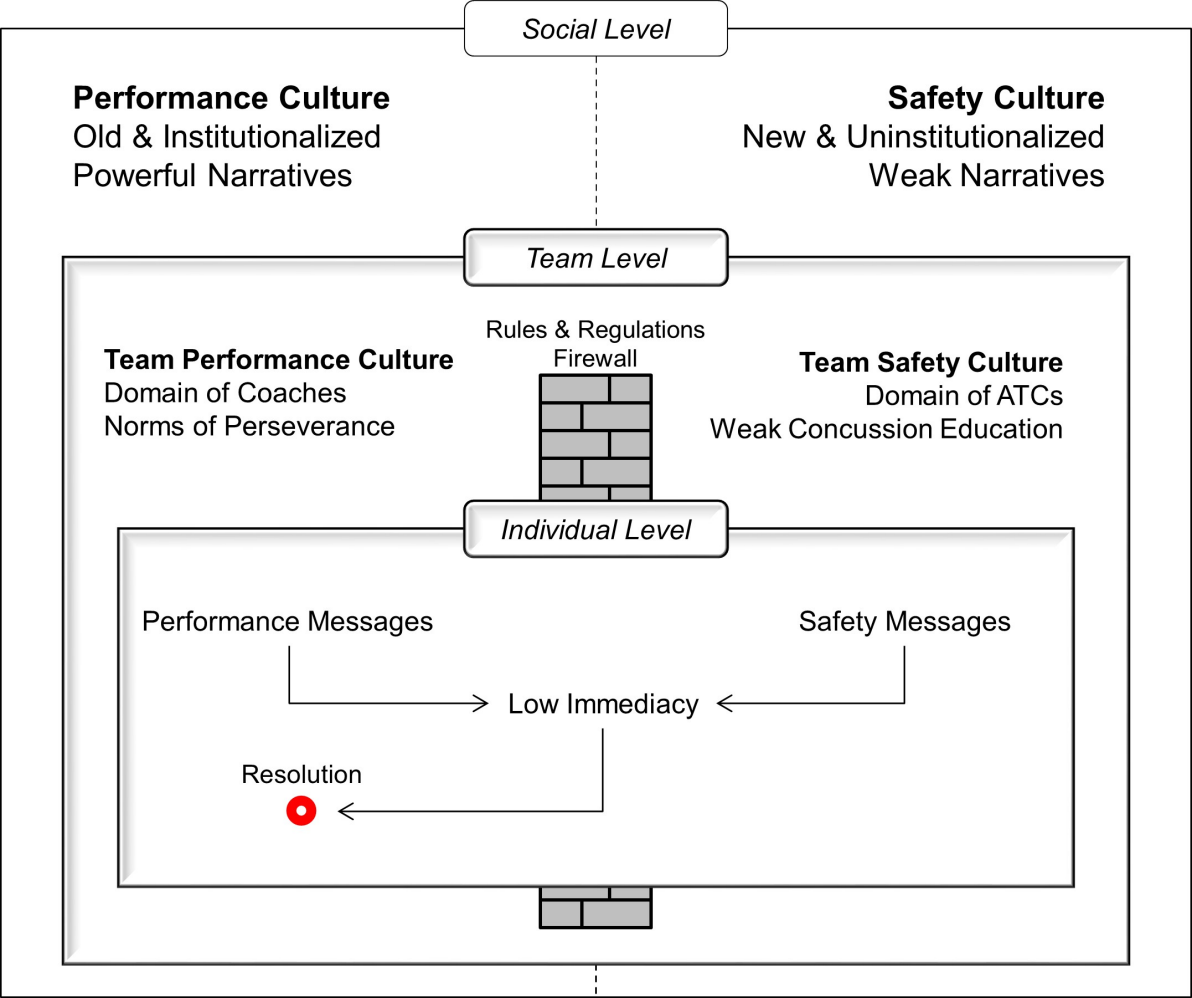
Influences of three socio-ecological levels on athlete reporting decisions.

Our integrated findings paint a picture (see Fig 2) in which athletes are accountable to two conflicting sets of organizational values, one focused on performance and another focused on safety. The performance values are the domain of coaches and are supported by a deeply institutionalized system of cultural narratives that promote toughness, masculinity, perseverance, and opportunity for those who adhere to these ideals. Safety is the domain of ATs (and medical staff) and is supported by a Need for Safety narrative that is new and not deeply institutionalized.

Two cultural narrative systems contextualize two aspects of team culture which produce mixed messages for the athlete. Because of low immediacy, these are resolved in favor of performance and non-reporting of severe head impacts. Athletes are therefore faced with competing value systems that create competing messages: On one hand perform like a warrior, including when you are injured, but on the other hand take care to protect yourself from concussions. Because athletes perceive concussion as a low-immediacy problem where they have little self-efficacy, the conflicting messages can be most easily resolved by elevating performance considerations over safety considerations. We hypothesize that this is likely a significant factor in failure to report SHIs.

Fortunately, this scenario also points to opportunities for better understanding and increasing SHI reporting behavior. First, future research should empirically test this multi-level mechanism for SHI under-reporting. It would look for evidence of performance/safety conflict, and test whether vested interests, organizational culture, and narrative enable its resolution in favor of performance considerations, as proposed. In particular, research should take a closer look at the role of perceived pressure from teammates and coaches.

Second, educational efforts can be made more frequent and memorable, can be evaluated, and can be more closely tailored to male and female athletes in specific sports. Our results indicate two directions such efforts might take. First, they would target immediacy by linking concussions not only to athletes’ health but to their potential impact on immediate fitness-to-play and team success, weakening the contradiction between performance and safety. For example, research shows that playing with concussion significantly increases an athlete’s risk of sustaining a musculoskeletal injury [69], an immediate risk about which athletes may not be aware. Second, in college sports, coaches play an essential role in defining team culture [46,70]. Yet we found that organizational structures have been put in place to limit coaches’ involvement in concussions, and this has diminished their support of educational efforts. Coaches can take a more active role in supporting educational efforts, thereby easing athletes’ fears that reporting SHIs may diminish their standing.

Finally, future research should also compare vestedness between athletes who have sustained diagnosed concussions to those who have not. We found that athletes with diagnosed concussions in their career tended to perceive higher risk, be more protective, and know the recovery process better. Incorporating their stories and experiences into educational efforts could make them more effective.

### Limitations

This study has a few limitations that deserve mention. One is that athletes were recruited on an availability basis, rather than at random. This was unavoidable because of the limitations placed on us by participating athletic programs. It is possible that the recruiting procedures introduced bias into the sample such that the results do not reflect the true values for the whole population; however, we saw no evidence of this.

A second limitation is that the study included basketball because it is the only sport where all Pac-12 schools have both men’s and women’s teams, affording the opportunity to systematically compare male and female attitudes within the same sport. Basketball does not involve intentional contact and carries a lower concussion risk than some of the other sports we studied, but it is still significant (3.89 and 5.95 incidents per 1000 athlete exposures for men and women, respectively [56]). It is possible that athletes in this sport biased results because they have a lower awareness of concussion risks.

A third limitation that is present in any qualitative study is potential bias in interpretation of results. We mitigated this by using multiple coders and a rigorous procedure for applying Schein’s framework and identifying themes. We also reviewed interpretations with an athletic trainer who was not involved in the analysis to guard against bias.

A final potential limitation identified by a reviewer is that some of the information in the qualitative analysis comes from reports of the athletes’ own experience, and some comes from athletes’ observation of their teammates’ experience. We do not believe this is a significant problem because observation of others is a key means by which organizational culture is propagated. For example, if an athlete observes a teammate having a SHI, appearing impaired, but continuing to play, he or she might conclude this is normative behavior for the team and act accordingly. We avoided using the other-observation data to draw conclusions about how athletes experience of concussion themselves, and instead focused on what the athletes take away from these observations in terms of organizational culture.

## Supporting information

S1 Table Descriptive coding categories to classify organizational artifacts. (DOCX)

S2 Table Descriptive coding categories to classify organizational values/norms. (DOCX)

S3 Table Survey items used in the study. (DOCX)
