# Supplementary material for: Socioecological influences on concussion reporting by NCAA Division 1 athletes in high-risk sports
Source: PLoS One. 2019 May 8;14(5):e0215424. doi: 10.1371/journal.pone.0215424 (PMC6505740; doi:10.1371/journal.pone.0215424)
Supplement: S1 Table — (DOCX) [file pone.0215424.s001.docx]

| **Category** | **Definitions** | **Number of Interviews** | **Number of Units** |
| --- | --- | --- | --- |
| Team identity & dynamics | Description of how the team sees itself, how members interact, how they orient toward their goals, how they build and maintain relationships. | 76 | 314 |
| Team composition | Description of how many senior or freshman players play in a specific team. | 25 | 38 |
| Team bonding rituals | Stories about teammate interaction. Do they make an effort within the team to bond everyone together? | 19 | 31 |
| Slogans | Slogans that orient the team in a particular way, or privilege a certain behavior or approach. | 44 | 68 |
| Legal/procedural requirement | Mentions of official policy: Preseason impact testing, SCAT testing, IMPACT testing, sign acknowledge forms, coaching staff training every year. | 88 | 338 |
| Education | Formal and informal efforts to educate athletes about concussion. | 82 | 238 |
| Effectiveness of educational materials | How do they think about the effectiveness of educational materials? | 49 | 92 |
| Other | Discussion or relevant artifacts not fitting the above categories. | 13 | 34 |
