# Supplementary material for: Socioecological influences on concussion reporting by NCAA Division 1 athletes in high-risk sports
Source: PLoS One. 2019 May 8;14(5):e0215424. doi: 10.1371/journal.pone.0215424 (PMC6505740; doi:10.1371/journal.pone.0215424)
Supplement: S2 Table — (DOCX) [file pone.0215424.s002.docx]

| **Category** | **Definitions** | **Number of Interviews** | **Number of Units** |
| --- | --- | --- | --- |
| Performance | An emphasis of being the highest performing team in PAC-12 is indicative of valuing winning over student health. | 33 | 52 |
| Toughness and handling adversity | Team norms of playing through pain because we want to make it. Team norms of playing through adversity or toughing it out. | 37 | 68 |
| Open communication | Staff accommodate students’ situations to articulate support of player. | 48 | 121 |
| Barriers to communication | Lack of accommodation coming from athletic trainers and coaches makes students fearful or worried about reporting to coaches because they are likely to be perceived as lying about symptoms or slacking. | 31 | 96 |
| Persuasive strategies used with athletes | Team-level appeal to students’ intrinsic motivation to keep playing: Handle sooner, return sooner, gain more playing time. | 24 | 44 |
| Internal communication supporting concussion management | The degree to different nodes within organizations actually communicate to each other. | 51 | 196 |
| Staff competence | The degree to which coaches or students value athletic trainers’ knowledge, expertise and skills. | 40 | 83 |
| Friction/collapse of system/conflict | Situations where coach and players says they’re ready to play but trainer says no. | 23 | 54 |
| Trust | Coaches trust athletic trainers to make the judgment call; Athletic trainers trust players are OK or rely on self-reporting if not witnessing the incident. Students trust athletic trainers’ judgments. | 56 | 159 |
| Separation of responsibility or decision making | Culture expects ATCs and physicians to take full responsibility in determining if a player should stay in game or practice. | 41 | 113 |
| Appreciation of employees | The degree to which organizational members publicly praise ATCs or coaches. | 1 | 1 |
| Perceived norm of reporting injury | The degree to which an athlete perceives the norm of reporting and responding to injury, both concussion and non-concussion. | 39 | 175 |
| Peer pressure | Reported actual or perceived pressure by peers to avoid reporting concussion symptoms or following protocols. | 11 | 14 |
| Staff pressure | Reported actual or perceived pressure by coaching or athletic training staff to avoid reporting concussion symptoms or following protocols. | 20 | 35 |
| Family pressure | Reported actual or perceived pressure by family to avoid reporting concussion symptoms or following protocols. | 10 | 15 |
| Self pressure | Reported actual or perceived pressure by athletes themselves to avoid reporting concussion symptoms or following protocols. | 36 | 60 |
| Other | Discussion of relevant values and norms not fitting the above categories | 45 | 203 |
