# Supplementary material for: Socioecological influences on concussion reporting by NCAA Division 1 athletes in high-risk sports
Source: PLoS One. 2019 May 8;14(5):e0215424. doi: 10.1371/journal.pone.0215424 (PMC6505740; doi:10.1371/journal.pone.0215424)
Supplement: S3 Table — (DOCX) [file pone.0215424.s003.docx]

| Class | Variable | Question Text |
| --- | --- | --- |
| Vestedness | | |
|  | Salience | - I often think about severe head impacts.^a^ - I am concerned about the health consequences related to severe head impacts.^a^ - It is easy for me to recall information I've learned about severe head impacts.^a^ - Severe head impacts are an important issue for me.^a^ - Generally, I care about health consequences related to severe head impacts.^a^ |
|  | Certainty | - Severe head impacts are likely to affect me.^a^ - I am certain that health consequences related to severe head impacts will influence my life in a significant way.^a^ - The odds are great that I will be affected by severe head impacts.^a^ - I expect severe head impacts to be a major factor in my life.^a^ - Severe head impacts are something that could definitely play a role in my future.^a^ |
|  | Immediacy | - Health consequences will occur soon after experiencing severe head impacts.^a^ - I do not have much time before I experience negative health consequences from severe head impacts.^a^ - Health consequences related to severe head impacts are not too far off in the future.^a^ - It will not be long until I will see the health consequences of severe head impacts.^a^ - I should be concerned about ­health consequences related to severe head impacts right now.^a^ |
|  | Self-efficacy | - I am capable of responding to health consequences related to severe head impacts.^a^ - I have the ability to reduce the health consequences of severe head impacts.^a^ - It would be easy for me to minimize my risk from severe head impacts when participating in [sport].^a^ - I have enough knowledge to be able to minimize my risk from severe head impacts I might suffer while participating in [sport].^a^ - I believe I can succeed at minimizing my risk from severe head impacts.^a^ |
|  | Response-efficacy | - [University]'s concussion education and treatment programs help minimize the negative health consequences related to severe head impacts.^a^ - [University]'s concussion education and treatment programs are a practical way to reduce any distress caused by severe head impacts.^a^ - [University]'s concussion education and treatment programs can help me respond to the health consequences of severe head impacts.^a^ - [University]'s concussion education and treatment programs are an effective way to reduce long term harm caused by severe head impacts.^a^ - It makes sense to me that [university]'s concussion education and treatment programs are recommended as an approach to severe head impacts.^a^ |
| Risk Perception | | |
|  | Perceived risk | - I am susceptible to severe head impacts.^a^ - I am at risk of suffering severe head impacts while participating in [sport].^a^ - I am vulnerable to severe head impacts while participating in [sport]-related activities.^a^ - There is a high possibility that health consequences related to severe head impacts can affect me.^a^ - I am sensitive to the health effects of severe head impacts.^a^ |
| Head injury | | |
|  | Concussions | - Have you ever been formally diagnosed with a concussion due to playing or practicing [sport]?^b^ - Were you ever forced to stop playing?^b^ - For the most recent time you were forced to stop playing: how many days were you unable to play?^c^ |
|  | Severe Head Impacts | - Have you ever suffered a severe head impact while playing or practicing [sport] that was not diagnosed as a concussion?^b^ - How many times?^b^ - Were you forced to stop playing?^c^ - For the most recent time you were forced to stop playing: how many days were you unable to play?^c^ |
|  | Sex | What is your sex?^d^   - Male - Female |
|  | Age | How old are you in years?^c^ |
|  | Ethnicity | What is your ethnicity?^d^   - American Indian/Alaskan Native - Asian - Black/African American - Hispanic / Latino/a - Native Hawaiian/Pacific Islander - White / Caucasian - Other |
|  | University | For what university do you play?^d^   - (List of participating universities) |
|  | Sport^d^ | What sport to you play?^d^   - Basketball - Lacrosse - Field Hockey - Football - Soccer - Wrestling |
|  | Lifetime years in sport^c^ | For how many years have you participated in [Previously selected sport]?^c^ |
|  | University years in sport^c^ | For how many years at [Previously reported University] have you participated in [Previously selected sport]?^c^ |
|  | Scholarship status^e^ | Please indicate your scholarship status^d^   - “Full-Ride scholarship - “Partial Scholarship" Athlete - "Walk-on" Athlete |

Items in brackets are dynamically generated depending on demographic responses.

^a^Seven potential scale responses, anchored by “Strongly Disagree” – “Strongly Agree.”

^b^Yes/No response

^c^Numerical fill-in

^d^Single-choice checkbox, with options show in bullets
